# What if all the Follicles were already Ruptured at the Time of Oocyte Pick-up in an IVF/ICSI Cycle?

**DOI:** 10.1055/s-0042-1745793

**Published:** 2022-06-27

**Authors:** Tayfun Cok

**Affiliations:** 1 Adana Research and Education Centre, Department of Obstetrics and Gynecology, School of Medicine, Baskent University, Adana, Turkey

In vitro fertilization (IVF) and intracytoplasmic sperm injection (ICSI) treatments are the assisted reproductive technologies (ARTs) with the highest success rate. In vitro fertilization and ICSI treatment have a high cost for couples including both the technology and the drugs used for ovulation induction. It is common to face prematurely ruptured follicles at the time of oocyte pick-up (OPU). The premature rupture of the follicles can happen due to many reasons such as idiopathic rupture of the follicles before the expected ovulation time, particularly in poor responder patients and inadvertent timing of ovulation trigger injections due to misunderstanding of the drug regimen. Also, it may be due to a delayed arrival to the ART clinic for OPU. There is a lack of data in the literature to salvage the IVF/ICSI cycles in such a situation after the use of high-cost injections for ovulation induction.

As we know from our basic knowledge about menstrual cycle physiology, after the rupture of the follicle, oocytes in the follicular fluid may be absorbed by the fallopian tubes with a negative pressure formed by the peristaltic movements of the tubes, directly or after spillage into the Douglas pouch. Therefore, following the rupture of the follicle, oocytes may be present in the peritoneal cavity, especially in the Douglas pouch, in the fallopian tubes, or in the uterus. 1

Here, we present a 36-year-old patient in an ICSI and preimplantation genetic testing for aneuploidy (PGT-A) cycle. The couple had a diagnosis of unexplained infertility after 3 years of subfertility and a diagnostic work-up. She was administered 300 IU follitropin alpha (Gonal-F; Merck, Istanbul, Turkey) daily starting on the 2 ^nd^ day of her menstrual cycle and dydrogesterone 10 mg (Duphaston; Abbott B.V., Netherlands) was added twice a day on the 5 ^th^ day of stimulation and was continued until the day of ovulation trigger to prevent premature ovulation. After 10 days of stimulation, 8 follicles ranging from 15 mm to 21 mm were observed and ovulation was triggered with choriogonadotropin alfa 500 micrograms (Ovitrelle; Merck, Istanbul, Turkey), and oocyte pick-up was scheduled 36 hours after the ovulation trigger. The patient misunderstood the time of choriogonadotropin alfa injection for ovulation trigger at 11:30 pm as at 11:30 am. When she arrived at our ART clinic, she was on the 45 ^th^ hour after ovulation trigger. Her transvaginal ultrasound examination revealed that all the follicles were ruptured, and there was some free fluid in the Douglas pouch. After a discussion of the situation with the patient, informed consent was given by the patient. Under sedoanalgesia, a single lumen 17G OPU needle (Cook Medical, Bloomington, IN, USA) was administered with the guidance of transvaginal ultrasound into the Douglas pouch, and the free fluid was aspirated. Two mature oocytes were found after the microscopic evaluation of the free fluid. Then, an intrauterine insemination catheter (Wallace Intrauterine Insemination Catheters; CooperSurgical Inc., Trumbull, CT, USA) was administered through the uterine cervix into the uterine cavity. The uterine cervix was clamped with a single tooth tenaculum forceps to prevent reflux. Then, 60 ml of Quinn Advantage Medium with HEPES (SAGE Media; CooperSurgical, Trumbull, CT, USA) was given intracavitaryly and the patient was positioned in a reverse Trendelenburg position. Free fluid newly formed in the Douglas pouch was also aspirated and, after the microscopic evaluation, three more mature oocytes were collected. After the ICSI procedure of the five metaphase II oocytes, four embryos were obtained, one of which reached the blastocyst stage. Preimplantation genetic testing for aneuploidy (PGT-A) was done to one embryo and one grade 5AB euploid embryo was obtained. 2

Although aspiration of Douglas pouch has been suggested in such situations, 3 we performed a novel approach that has never been mentioned in the literature. Aspiration of the free fluid in the Douglas pouch may be feasible when there is a small time gap between the ovulation and the OPU, but when this time gap increases, as in our case, the possibility of oocytes being in the fallopian tubes increases, which may cause Douglas pouch aspiration to be ineffective. Instead of canceling the whole ICSI/PGT-A cycle in such a desperate situation, after the consent of the patient, we searched the uterine cavity and fallopian tubes for oocytes assuming that the oocytes should be absorbed by the tubes by the time of the arrival of the patient to the clinic on the 45 ^th^ hour. We obtained a euploid blastocyst at the end of the treatment. It is not known if this procedure has any detrimental effect on the endometrium and on embryo implantation; if used in a fresh IVF/ICSI cycle, the embryo transfer may be postponed to the thawing cycle after freezing all embryos. However, as this treatment cycle was a progestin-primed ICSI/PGT-A cycle, all the embryos were going to be frozen, so we did not have any concern about the endometrium.

We suggest that any rescue intervention for oocytes should be performed according to the estimated timing of the follicule rupture in an IVF/ICSI cycle and to the presentation of the patient at the clinic.
